# No Benefit from Hydroxyurea Pre-Treatment in Frontline Chronic Myeloid Leukemia Therapy and Evidence of Quantitative Changes in the *BCR::ABL1* Transcript Level

**DOI:** 10.3390/ijms26051840

**Published:** 2025-02-21

**Authors:** Fabio Stagno, Stefania Stella, Salvatore Leotta, Santino Caserta, Silvia Rita Vitale, Uros Markovic, Cristina Tomarchio, Giuseppe Mirabile, Sabina Russo, Michele Massimino, Paolo Vigneri, Livia Manzella, Francesco Di Raimondo, Alessandro Allegra

**Affiliations:** 1 Hematology Section, A.O.U. Policlinico “G. Martino”, University of Messina, 98100 Messina, Italy; santino.caserta@polime.it (S.C.); dottoressarusso20@gmail.com (S.R.); aallegra@unime.it (A.A.); 2 Department of Clinical and Experimental Medicine, University of Catania, 95123 Catania, Italy; stefania.stella@unict.it (S.S.); silviarita.vitale@gmail.com (S.R.V.); cristina.tomarchio@hotmail.it (C.T.); vigneripaolo@gmail.com (P.V.); manzella@unict.it (L.M.); 3 Center of Experimental Oncology and Hematology, A.O.U. Policlinico “Rodolico-San Marco”, 95123 Catania, Italy; michedot@yahoo.it; 4 Hematology and BMT Unit, A.O.U. Policlinico “Rodolico-San Marco”, University of Catania, 95123 Catania, Italy; leotta3@yahoo.it (S.L.); urosmarkovic09041989@gmail.com (U.M.); diraimon@unict.it (F.D.R.); 5 Department of General Surgery and Medical-Surgical Specialties, University of Catania, 95123 Catania, Italy; 6 University Oncology Department, Humanitas Istituto Clinico Catanese, 95045 Catania, Italy

**Keywords:** chronic myeloid leukemia, CML, CML therapy, hydroxyurea, *BCR::ABL1*, *BCR::ABL1/ABL1* transcript, TKI, HU

## Abstract

Hydroxyurea (HU) cytoreduction is usually administered to patients with chronic myeloid leukemia before starting any tyrosine kinase inhibitors (TKIs) therapy. However, up to date, there is no evidence of any benefit of hydroxyurea pre-treatment. Conversely, evidence has been provided on both the prognostic significance of the quantitative assessment of *BCR::ABL1* expression at diagnosis and the individual decline of the *BCR::ABL1* slope. In this view, we assumed that any kind of treatment administered before a confirmed diagnosis of chronic myeloid leukemia might change the amount of *BCR::ABL1* transcript levels. To this purpose, we evaluated leukocyte counts and *BCR::ABL1* quantitative expression either at diagnosis (baseline and no therapy) and on day 7 and day 14 of treatment in a cohort of 45 unselected patients with newly diagnosed chronic myeloid leukemia in the chronic phase. After informed consent, 21 of them received HU cytoreduction for 14 days before starting TKI treatment (HU group), whereas the other 24 patients received frontline TKI therapy without HU pre-treatment (TKI group). Our findings showed that: (i) there is no benefit from HU cytoreduction in patients affected with chronic myeloid leukemia before starting treatment with TKIs; (ii) any kind of therapy administered before a confirmed diagnosis of chronic myeloid leukemia might change the amount of *BCR::ABL1* expression levels.

## 1. Introduction

The treatment of chronic myeloid leukemia (CML) has been drastically changed by the approval of tyrosine kinase inhibitors (TKIs) [1]. CML is now managed as a chronic disease requiring both long-term treatment and close molecular monitoring in most patients. This unprecedented improvement in CML clinical management and therapy has been recognized by a panel of leukemia experts who, on behalf of the European Leukemia Net (ELN), have first recommended Imatinib (IM) for the frontline treatment of the disease [2,3,4,5]. However, despite groundbreaking results, approximately 40–50% of patients with CML eventually discontinued IM because of intolerance to the drug or unsatisfactory responses [6]. Addressing these issues, second-generation (2G) TKIs have also been approved for the frontline treatment of CML, since they achieve both faster hematologic and cytogenetic responses and deeper reductions in *BCR::ABL1/ABL1* transcript levels [7].

Before the clinical advent of TKIs, *α*-interferon (IFN) or IFN-based therapies were the treatment of choice for CML, and hydroxyurea (HU) also played a major clinical role [8,9]. Currently, pre-treatment with HU is usually administered as cytoreductive therapy to patients with CML in the chronic phase before starting TKIs’ treatment, particularly in those showing high white blood cell (WBC) counts. Moreover, some clinical protocols allow a period of HU therapy up to three months before starting the protocol schedule.

Nevertheless, evidence of any benefit of HU cytoreduction has not been provided. On the other hand, evidence has been shown on both the prognostic significance of the quantitative assessment of *BCR::ABL1* expression at diagnosis [10] and the individual decline of the *BCR::ABL1/ABL1* slope [11]. Hence, we assumed that any kind of treatment administered before a confirmed diagnosis of CML might change the amount of *BCR::ABL1* expression. In this view, we evaluated WBC counts and *BCR::ABL1* quantitative level either at diagnosis and on day 7 and day 14 of treatment in a cohort of patients with newly diagnosed *BCR::ABL1*-positive CP-CML treated with or without cytoreduction with HU before frontline treatment with TKIs. Furthermore, we also investigated whether cytoreduction with HU might influence the achievement of an early molecular response (EMR) of a major molecular response (MR^3^), as well as an optimal clinical response.

## 2. Results

Overall, 45 patients with newly diagnosed CP-CML (30 males and 15 females, median age 61.7 years) were evaluated, and their clinical characteristics are summarized in Table 1. Patients were stratified into two groups according to having received cytoreduction with HU (the HU group) or not (the TKI group). Of them, 21 (46.6%) received a preliminary cytoreductive treatment with HU (500 mg os bis in die) for a period of fourteen days, whereas 24 (53.4%) patients were treated directly with TKIs (IM, Dasatinib, Nilotinib) at conventional doses. Briefly, PB samples were collected at diagnosis (baseline data with no therapy) and on day 7 and day 14 of treatment in order to analyze white blood cell (WBC) counts and *BCR::ABL1/ABL1* quantitative levels between the two groups.

**Figure 1:**
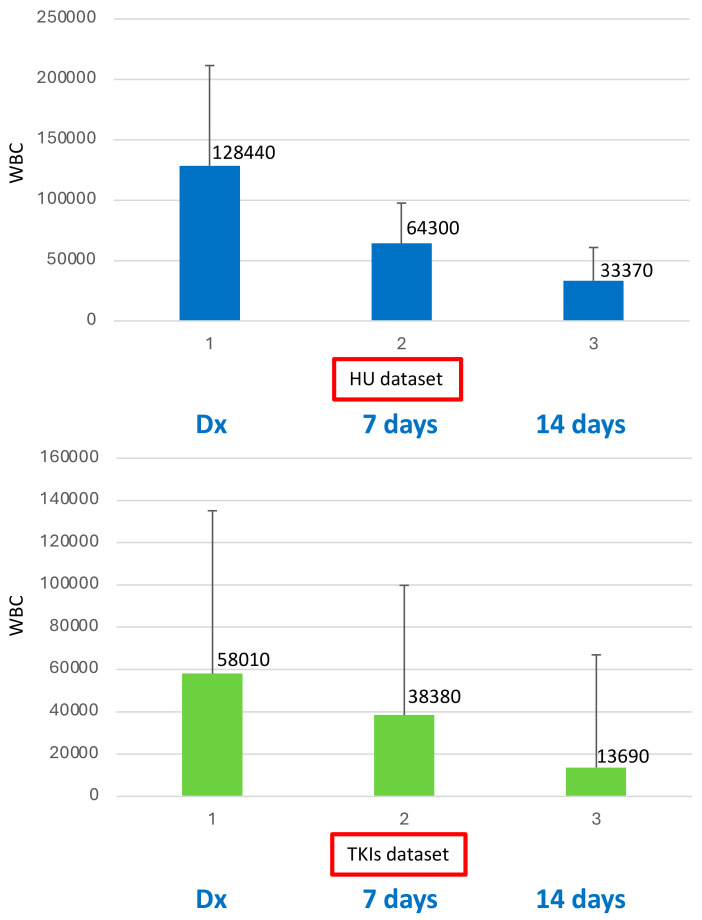
WBC counts detected at diagnosis and on day 7 and day 14 of treatment for the HU and TKI groups. Baseline WBC medians showed a difference between the two dataset groups (HU vs. TKIs) for HU (*p* = 0.02); no difference was detected on both day 7 and day 14 between the two dataset groups (HU vs. TKIs) (*p* = 0.37 and *p* = 0.36, respectively). Legend: 1 (Baseline/Diagnosis); 2 (day 7); 3 (day 14).

As expected, WBC medians at diagnosis (baseline) showed a statistical difference between the two groups (HU-WBC: 128,440, range 26,500 ± 364,254; vs. TKI-WBC: 58,010, range 21,180 ± 368,380) in favor for the HU group (*p* = 0.02). Conversely, no difference was detected when we evaluated the WBC count on day 7 (HU-WBC 64,300 vs. TKI-WBC 38,380; *p* = 0.37) and on day 14 (HU-WBC 33,370 vs. TKI-WBC 13,690; *p* = 0.36) between the two groups (Figure 1). 

**Figure 2:**
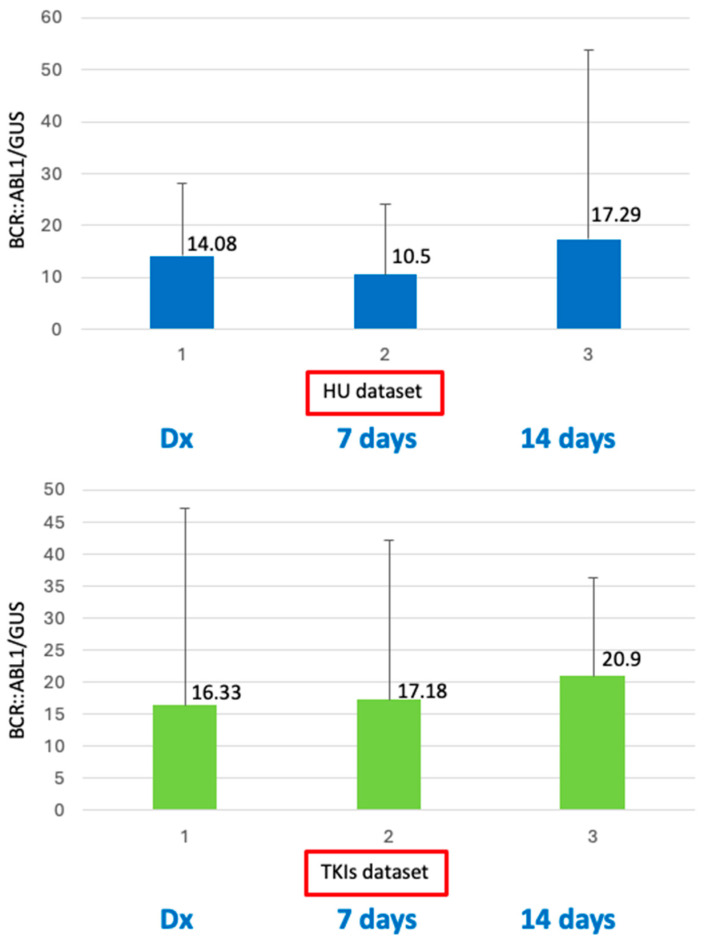
Difference in *BCR::ABL1/GUS* % transcript levels between the HU and TKI groups. No statistical differences were detected between HU vs. TKI groups. At diagnosis, *p* = 0.2; on day 7, *p* = 0.1; on day 14, *p* = 0.6. Legend: 1 (Baseline/Diagnosis/Dx); 2 (day 7); 3 (day 14).

Similarly, *BCR::ABL1/GUS* transcript levels were measured from PB samples drawn at the above-prefixed time points (diagnosis and day 7 and day 14 of treatment) with the purpose of evaluating the impact of HU cytoreduction and to observe any change in the amount of *BCR-ABL1* expression level. *BCR::ABL1/GUS^IS^* median transcript levels at diagnosis (baseline) were 14.08% for the HU group and 16.33% for the TKI group (*p* = 0.2). On day 7, the median *BCR::ABL1/GUS^IS^* was 10.50% for the HU group and 17.18% for the TKI group (*p* = 0.1), and on day 14, the median *BCR::ABL1/GUS^IS^* was 17.29% for the HU group versus 20.90% for the TKI group (*p* = 0.6) (Figure 2).

Furthermore, when we analyzed the patients who achieved an early molecular response (EMR) at three months, we found no difference (*p* = 0.2) between the two groups according to *BCR::ABL/ABL1^IS^* levels (*p* = 0.2; Table 2), although a trend in favor of the TKI group was shown.

**Figure 3:**
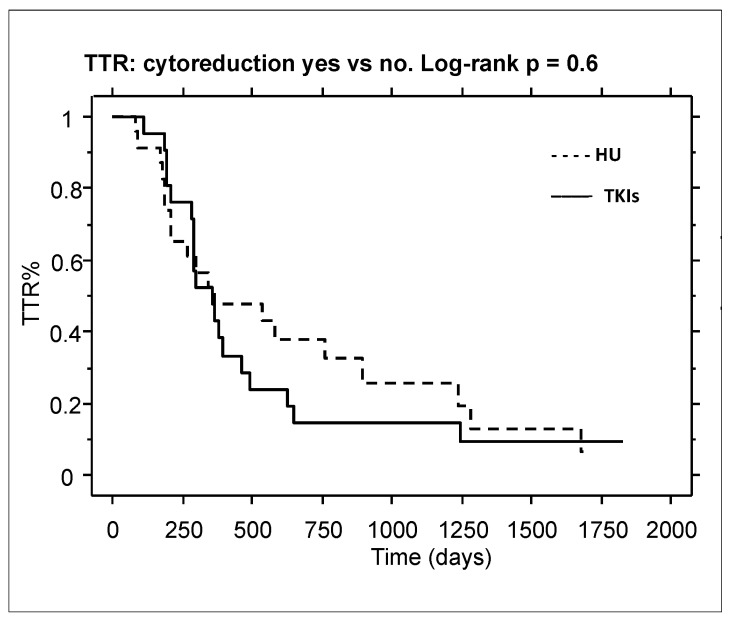
Time to Treatment Response (MR^3^). No differences were detected between the HU group vs. the TKI group. Median TTR was 359 (109–1825) and 369 (84–1687) days, respectively, without significant difference (*p* = 0.6) (359 vs. 369 days, *p* = 0.6). HU = HU group; TKI = TKI group.

Finally, we evaluated if HU cytoreduction, although for a short period, played a possible role in obtaining an MR^3^ (Time To Treatment Response, TTR). Again, we observed no difference between the HU and TKI groups (*p* = 0.6; Figure 3).

## 3. Discussion

Treatment with HU, an inhibitor of the ribonucleotide reductase, has been revealed to be useful in the past in managing CML and has also been used either as frontline therapy [12] or associated both with IFN therapy [8] and IM treatment [13]. However, although the CML study I suggested that IFN in association with HU was superior to IFN alone [8], a successive German investigation demonstrated no benefit of using HU in combination with IM over IM monotherapy [13].

Currently, pre-treatment with HU is usually administered as a cytoreductive therapy to patients with CML before starting any TKI treatment, particularly in those showing hyperleukocytosis [7]. However, until now, there is no clear evidence of any benefit of HU pre-treatment in newly diagnosed CP-CML. In this view, we investigated the possible impact of a short course of cytoreduction with HU both on WBC counts and on quantitative *BCR::ABL1* level changes, if any, either at diagnosis and on day 7 and day 14 of treatment in a cohort of patients with newly diagnosed *BCR::ABL1*-positive CP-CML treated with or without cytoreduction with HU before frontline treatment with TKIs. Our findings showed that there is no need for cytoreduction with HU therapy in patients affected with CML before starting treatment with TKIs since we found no statistical difference in WBC cytoreduction both on day 7 and 14 between the two groups (HU vs. TKI, *p* = 0.37 and *p* = 0.36, respectively). Moreover, patients with CP-CML accrued in the HU group exhibited a median baseline high WBC count as compared to the TKI group (*p* = 0.02), mostly because of clinician choice. Therefore, we provide evidence that HU cytoreduction has no additive value.

We next wanted to verify if any kind of therapy administered before a confirmed diagnosis of CML might change the amount of *BCR::ABL1* expression levels. Published data support both the prognostic significance of the quantitative assessment of *BCR::ABL1* expression at diagnosis and the individual decline of the *BCR::ABL1* slope [10,11]. Furthermore, it must be outlined that high *BCR–ABL/GUS^IS^* expression is probably indicative of higher amounts of *BCR::ABL1* transcripts within each leukemic cell, that is, a well-established sign of disease progression in CML [10,14]. In this experiment, we found that median *BCR::ABL1/GUS^IS^* transcript levels at diagnosis (baseline) were 14.08% for the HU group and 16.33% for the TKI group (*p* = 0.2). On day 7, median *BCR::ABL1/GUS^IS^* showed a decline to 10.50% for the HU group and a slight and not significant increase to 17.18% for the TKI group (*p* = 0.1), whilst on day 14, the median *BCR::ABL1/GUS^IS^* was 17.29% for the HU group versus 20.90% for the TKI group (*p* = 0.6). All these data demonstrate that any kind of therapy, either cytoreductive or target therapy as TKIs, administered before or after a confirmed diagnosis of CML might change the kinetics of the *BCR::ABL1* transcript.

Hence, we report evidence that any course of treatment, even short in time, might cause quantitative changes in the *BCR::ABL1* expression and might also suggest a different impact against CML clones driven by different therapies. Until recently, some trials allowed patients with CML to be assigned to a TKI treatment in a pre-randomization phase [15,16]. Therefore, caution must be exerted when evaluating *BCR::ABL1* transcript levels at diagnosis after some therapy.

Lastly, we wanted to investigate if HU pre-treatment might influence the achievement of an EMR at three months or the time to obtain an MR^3^, as well as an optimal clinical response. Overall, we found no difference in the achievement of both an EMR (*p* = 0.2) and an MR^3^ (*p* = 0.6) between the two groups according to *BCR::ABL1/ABL1^IS^* levels. These last two clinical results also agree with those of Kockerols CCB and colleagues who, in a larger cohort of patients with CML having the same HU median treatment duration, suggested that HU cytoreduction has no adjunctive value in achieving clinical responses in CML treatment [17]. Furthermore, short-pulse HU therapy has been shown to enhance erythroid progenitor cell differentiation and to modify the characteristics of CML leukemic stem cells [18]. In this view, we have previously demonstrated that high *BCR::ABL1* expression leads to increased proliferation and anti-apoptotic signaling in CD34+ committed leukemic progenitors, promoting also stem cell division [19]. Our intriguing observations implicate that according to *BCR::ABL1* levels at diagnosis, patients with CML should be subjected to different clinical management. Patients with CML showing higher *BCR::ABL1* levels at diagnosis would be exposed to a higher risk of developing disease progression caused by a higher proliferation rate, thereby inducing additional molecular alterations that increase the persistence of peripheral blast cells.

Moreover, it deserves to be mentioned that HU therapy is not recommended during pregnancy because of its teratogenicity [20].

## 4. Patients and Methods

We accrued prospectively a cohort of 45 consecutive patients with newly diagnosed CP-CML. All procedures were performed in accordance with the ethical standards of each institutional research committee and with the Declaration of Helsinki and its later amendments.

After informed consent, 21 patients received baseline preliminary therapy with HU (500 mg os bis in die) for 14 days before starting TKIs’ treatment (HU group), whereas the other 24 patients received conventional frontline TKIs therapy without HU pre-treatment (TKI group) always at baseline (Supplementary Materials). No specific criteria for administering HU cytoreduction were applied, and it depended on the clinician’s choice. However, most of the patients registered in the hospital emergency department and most of those showing hyperleukocytosis received HU therapy. Clinical and molecular responses were defined as previously reported [7].

The *BCR::ABL1* transcript, *ABL1* expression, and GUS transcript levels were measured from peripheral blood (PB) samples drawn at diagnosis and on day 7 and day 14 and then every three months using real-time PCR (qPCR) analysis as previously described [10]. *ABL1* was used as the reference gene at any time point. In addition, at diagnosis and on day 7 and day 14, *BCR::ABL1* expression was also measured by using *GUS* as a housekeeping gene, as it is a more appropriate reference gene for specimens expressing high *BCR::ABL1* [21]. All samples were processed for nucleic acid extraction in the Center of Experimental Oncology and Hematology of the A.O.U. Policlinico “Rodolico-San Marco”, as previously described [22]. Real-time PCR (Q-PCR) determinations for *BCR::ABL1/ABL1* and *BCR::ABL1/GUS* were converted to the international scale (IS) [10,23,24,25] and were considered of appropriate quality only in the presence of no less than 24,000 *GUS* copies or 10,000 *ABL1* copies, as previously reported [24].

In brief, *BCR::ABL1* transcripts were measured from PB samples drawn at diagnosis and on day 7 and day 14 and then every 3 months thereafter using RQ-PCR. Samples were subjected to RQ-PCR using the TaqMan platform and both beta-glucuronidase and ABL as reference genes. For samples collected at diagnosis and on day 7 and day 14, we used GUS, as it is the more appropriate reference gene for specimens expressing high levels of *BCR::ABL1*. For *BCR::ABL1* transcripts measured every three months, ABL1 was the only reference gene used (Supplementary Materials). *BCR::ABL1/GUS* and *BCR::ABL1/ABL1* ratios were reported on IS using a conversion factor (CF) calculated within the laboratory at the University Hospital Mannheim.

### Statistical Analysis

Patients’ clinical characteristics were compared by the c^2^-test for categorical variables and the Mann–Whitney U-test for continuous variables. The time interval between the starting date of therapy and the date of MR^3^ achievement was considered as time to response (TTR). Survival analysis was estimated by the Kaplan−Meier method and was assessed by the log-rank test. All tests were 2-sided, accepting a *p*-value ≤ 0.05 as significant. The Stat View software 5.01 package (Mountain View, CA, USA) was used for statistical analysis.

## 5. Conclusions

**Figure 4:**
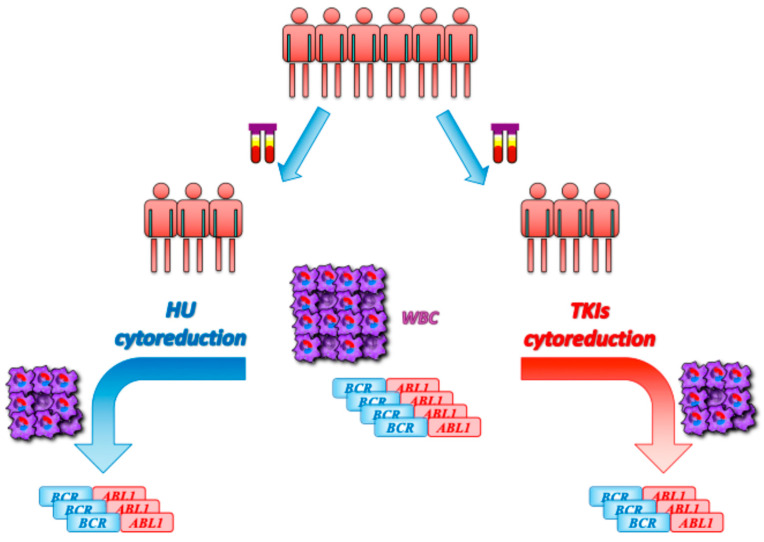
Graphical representation. HU pre-treatment in patients with CP-CML at diagnosis has no clinical cytoreductive benefit, and any kind of therapy administered before or after a confirmed diagnosis of CML might change the kinetics of the *BCR::ABL1* transcript. WBCs = white blood cells; HU = hydroxyurea; TKIs = tyrosine kinase inhibitors.

In conclusion, our findings reveal that HU pre-treatment in patients with CP-CML has no clinical cytoreductive benefit, and we demonstrate that any kind of therapy administered before a confirmed diagnosis of CML might change the amount of *BCR::ABL1* expression levels (Figure 4).

## Figures and Tables

**Table 1:** Patients’ clinical characteristics.

	HU	TKIs
**Patients no.**	21	24
**Age, median years (range)**	58 (18–79)	64.5 (17–83)
**Sex (M/F)**	15/6	15/9
**WBC × 10^9^/L, median (range)**	128.44 (26.5–197)	58.01 (24.46–368.38)
**Sokal risk**		
- **Low**	4	9
- **Intermediate**	11	13
- **High**	5	2
- **ND**	1	0
***BCR::ABL1* transcript**		
- **e13a2**	6	14
- **e14a2**	14	9
- **e13a2-e14a2**	1	1
***BCR::ABL1/GUS^IS^*, median % @ diagnosis**	14.08	16.33

**Table 2:** Early molecular response according to *BCR::ABL1/ABL^IS^* levels at three months.

*BCR::ABL1/ABL^IS^* < 10% @ 3 Months	HU Group	TKI Group	*p*
**Optimal response**	16	22	0.2
**Non optimal response**	5	2	
**ELN responders (EMR)**	76.1%	91.6%	

EMR = early molecular response.

## Data Availability

The original contributions presented in this study are included in the article/Supplementary Materials. Further inquiries can be directed to the corresponding author(s).

## Supplementary Materials

The supporting information can be downloaded at https://www.mdpi.com/article/10.3390/ijms26051840/s1.
