# Supplementary material for: No Benefit from Hydroxyurea Pre-Treatment in Frontline Chronic Myeloid Leukemia Therapy and Evidence of Quantitative Changes in the BCR::ABL1 Transcript Level
Source: Int J Mol Sci. 2025 Feb 21;26(5):1840. doi: 10.3390/ijms26051840 (PMC11899297; doi:10.3390/ijms26051840)
Supplement: Supplementary file 1 [file ijms-26-01840-s001.zip › ijms-3416573-supplementary.pdf]

Method for evaluation of *BCR::ABL1* transcripts

BCR::ABL1 transcripts were measured by using RQ-PCR (Applied Biosystems ABI 7500 Real Time PCR). We use primers and TaqMan probes (TaqMan dual-labelled hydrolysis probe) in conjunction with the TaqMan Universal Master Mix purchased from the same manufacturer.
The primers were used at concentration of 300nM (Forward: 5'-TCCGCTGACCATCAATAAGGA-3') (Reverse: 5' – CACTCAGACCCTGAGGCTCAA-3') while the probe at 200nM (FAM-CCCTTCAGCGGCCAGTAGCATCTGA-TAMRA).
The PCR condition was: 1. 95°C for 10 minutes; 2. 95°C for 15 seconds (50 cycles); 3. 60°C for 1 minutes.
For the standard curves we used five dilution points from *BCR::ABL1* 10^6 copies to *BCR::ABL1* 10^1 copies in duplicates (Ipsogen standards).
The threshold is used to determine the threshold cycle (Ct) and is typically set in the log-linear phase of the amplification curve. The threshold was set at constant 0.1 Ct and we obtained the curves slopes between −3,20 and −3,60.
Every time a batch of RNA extraction is performed, negative RT- and RT-qPCR blanks were included. In addition, we used different negative controls:

1. The water used for RNA elution from columns; or to resuspend RNA pellets;
2. Ethanol from RNA preparation;
3. RLT solution from each sample processing batch;
4. Non-Template Control (NTC, water).

In every plate we added also positive control, required to safeguard against false negative results due to failure in the amplification of the most common fusion subtypes. For this purpose, we added a positive control such as cDNA derived from mRNA extracted from K562 cells for the e14a2 fusion, to control specifically for the amplification/detection steps.
All samples were evaluated for amplification of a reference gene (*ABL1*) to verify quality following these stringent criteria. *ABL1* measurements were done in duplicate and single measurements are showed at least 10 000 *ABL1* copies per sample.

HU-group: patients' characteristics

PPN	Age years	Sex	Sokal risk	BCR::ABL1/GUS^{IS} % @ Diagnosis	BCR::ABL1/GUS^{IS} % @ 7 days	BCR::ABL1/GUS^{IS} % @ 14 days	WBC @ Diagnosis mmc	WBC @ 7 days mmc	WBC @ 14 days mmc	BCR::ABL1 transcript type
**1**	59	M	H	52,23	42,86	26,45	331140	120210	81190	e13a2
**2**	54	M	H	46,79	25,39	42,43	159000	93630	33370	e13a2
**3**	62	M	I	31,89	54,00	63,19	152360	110240	88370	e13a2
**4**	71	M	I	30,44	21,90	23,53	111110	79060	17440	e13a2
**5**	58	M	L	27,49	18,15	19,79	197000	119750	60380	e13a2
**6**	50	M	L	23,10	4,64	17,29	33350	29790	16330	e13a2-e14a2
**7**	37	M	I	21,45	18,56	21,92	91630	50700	36270	e14a2
**8**	51	M	H	21,39	20,24	19,11	93380	63910	53730	e13a2
**9**	43	M	I	16,58	22,93	170,74	106980	91460	29440	e14a2
**10**	64	F	I	15,63	7,40	9,82	128440	75640	46450	e14a2
**11**	58	M	I	14,08	17,86	20,63	152240	124550	104730	e14a2
**12**	77	F	H	13,91	11,29	8,37	77050	54790	41510	e14a2
**13**	18	M	I	12,68	6,69	9,08	188320	130570	81330	e14a2
**14**	65	M	I	10,37	10,50	24,82	105930	56490	20430	e14a2
**15**	38	M	L	7,46	1,25	5,33	166910	72850	25500	e14a2
**16**	59	M	L	7,24	3,89	9,07	26500	20900	12480	e14a2
**17**	34	M	H	4,70	7,67	8,90	364254	54900	33600	e14a2
**18**	61	F	I	4,18	0,20	3,32	139400	50730	27690	e14a2
**19**	75	F	I	3,79	5,02	7,43	70690	64300	27860	e14a2
**20**	79	F	I	2,13	0,69	4,48	174000	53200	6970	e14a2
**21**	55	F	ND	1,80	8,38	0,63	95000	23240	18330	e14a2

TKI-group: patients' characteristics

PPN	Age years	Sex	Sokal risk	BCR::ABL1/GUS^{IS} % @ Diagnosis	BCR::ABL1/GUS^{IS} % @ 7 days	BCR::ABL1/GUS^{IS} % @ 14 days	WBC @ Diagnosis mmc	WBC @ 7 days mmc	WBC @ 14 days mmc	BCR::ABL1 transcript type
**1**	67	F	I	143,68	56,44	18,36	32230	24970	14000	e13a2
**2**	83	M	L	65,46	42,89	63,81	128370	85460	48260	e13a2
**3**	71	M	L	54,49	104,61	23,20	28350	12900	7590	e13a2
**4**	17	M	L	47,67	74,04	27,68	24630	17250	5360	e13a2
**5**	53	F	I	40,68	29,91	19,61	56540	33440	7860	e13a2
**6**	65	M	L	36,14	34,15	30,79	72250	43660	9460	e13a2
**7**	55	M	I	29,38	31,37	33,89	107760	105000	28900	e13a2
**8**	68	M	L	28,20	27,00	33,68	26960	13650	9900	e13a2
**9**	73	F	I	23,7	18,16	15,22	57230	43320	16170	e13a2
**10**	64	F	I	22,32	21,34	42,12	36990	23190	9090	e13a2
**11**	75	M	I	19,93	26,35	33,12	91040	67380	18760	e13a2
**12**	80	M	I	16,4	17,08	7,08	58790	59950	34390	e14a2
**13**	48	M	H	16,27	17,28	21,00	139200	105060	49680	e13a2
**14**	51	M	I	14,48	10,79	20,90	129350	89560	22670	e14a2
**15**	34	M	L	13,29	15,46	26,19	170390	85260	13380	e13a2
**16**	26	M	L	9,52	10	ND	33260	14560	6090	e14a2
**17**	83	M	I	9,43	3,49	4,15	48550	21580	11590	e14a2
**18**	69	F	I	5,43	7,27	3,24	117760	108550	56660	e13a2
**19**	36	F	I	4,98	1,96	6,55	21180	17160	8040	e14a2
**20**	64	F	L	3,30	12,72	12,29	24460	12320	15020	e14a2
**21**	74	M	I	3,10	8,03	23,24	73500	31150	11710	e14a2
**22**	62	M	L	2,39	0,95	0,32	37520	25550	11270	e14a2
**23**	71	F	H	2,25	1,87	1,46	179670	107830	29200	e13a2-e14a2
**24**	41	F	I	0,79	2,91	1,18	368380	297060	270000	e14a2

Legend for HU-group and TKI-group: Male (M), Female (F); Sokal risk: High (H), Intermediate (I), Low (L), Not Determined (ND) because of previous splenectomy 5 years before diagnosis of CML.
